# Tendency in tip polarity changes in non-contact atomic force microscopy imaging on a fluorite surface

**DOI:** 10.3762/bjnano.16.72

**Published:** 2025-06-26

**Authors:** Bob Kyeyune, Philipp Rahe, Michael Reichling

**Affiliations:** 1 Institut für Physik, Universität Osnabrück, Barbarastraße 7, 49076 Osnabrück, Germany

**Keywords:** atomic resolution imaging, calcium fluoride surface, interaction force, non-contact atomic force microscopy (NC-AFM), tip change

## Abstract

We investigate the impact of tip changes on atomic-scale non-contact atomic force microscopy (NC-AFM) contrast formation when imaging a CaF_2_(111) surface. A change of the atomic contrast is explained by a polarity change of the tip-terminating cluster or by a polarity-preserving tip change via the re-arrangement of the foremost atoms. Based on the established understanding of the unique contrast patterns on CaF_2_(111), polarity-preserving and polarity-changing tip changes can be identified unambiguously. From analyzing a large set of images, we find that the vast majority of tip changes tend to result in negative tip termination. This analysis delivers hints for tip configurations suitable for stable imaging of CaF_2_(111) surfaces.

## Introduction

Non-contact atomic force microscopy (NC-AFM) [1] is a surface science tool that has been used to atomically resolve surfaces of semiconductor and insulator materials in real space with unprecedented spatial resolution [2–6]. Besides high-resolution imaging of molecular structures [7], NC-AFM has demonstrated its ability to identify sublattices of atomic surfaces [8–10]. In these studies, the knowledge of the tip’s atomic structure plays a vital role as the tip-terminating cluster uniquely interacts with the different surface atoms. At cryogenic temperatures, the use of functionalized tips such as as CO-terminated tips [6,11], Xe-terminated tips [12–14] and O-terminated Cu tips [15–17] has become the state-of-the-art for structure elucidation and identification of surface sites.

However, this approach is presently not feasible for measurements performed at room temperature as the required control over the tip termination is challenged by thermal motion. For room-temperature measurements, it is common practice to bring the tip apex in slight contact with the surface under investigation to form a tip cluster yielding atomic contrast [18]. As structure and chemical composition of the resulting tip-terminating cluster are not known, the understanding of contrast formation with non-functionalized tips has been developed over many years based on theoretical simulations of NC-AFM data for a variety of plausible tip models [9,19–22]. Through further endeavors, a qualitative distance-dependent approach involving electrostatic interactions and Pauli repulsion has recently been exemplified on CaF_2_(111) [10]. As a central result, gradual atomic contrast transitions as a function of the tip–sample distance have been introduced as criteria for identifying a positively and a negatively terminated tip [10]. Still, a crucial aspect in sublattice identification studies is to distinguish between contrast changes caused by a change of the tip-terminating cluster (i.e., a tip change) and a distance-dependent contrast evolution for a stable tip. Tip changes are inevitable in NC-AFM experiments with non-functionalized tips, especially as commonly used silicon tips are very reactive and readily pick up various entities. This particularly concerns the transfer of surface species to the tip when the tip is subject to intentional or unintentional contact with the surface. Furthermore, ambient species like native oxides, hydrogen ions, or residual water can adsorb on the tip apex during scanning. Additionally, the foremost tip atom may rearrange to minimize the tip surface energy in response to increasing tip–sample interaction forces.

**Figure 1:**
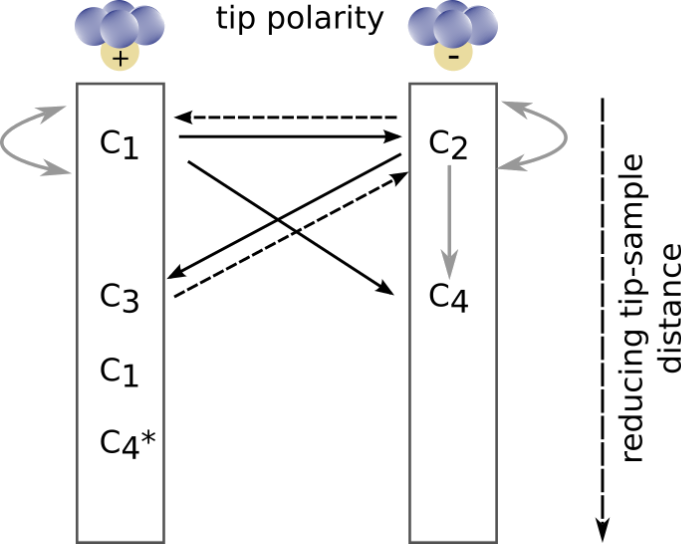
Distance-dependent contrast formation on CaF_2_(111) for positively and negatively terminated tips (vertical columns) as well as transitions between contrast modes due to tip changes. The contrast modes **C**_1_, **C**_3_, and **C**_4_* (**C**_2_ and **C**_4_) are assigned to a positive (negative) tip termination [10]. Solid black arrows indicate experimentally observed changes of the tip polarity, while grey arrows denote polarity-preserving tip changes. Dashed black arrows indicate tip changes that were observed but are excluded from the discussion herein.

Here, we perform an experimental investigation of tip changes during NC-AFM imaging of a CaF_2_(111) surface with non-functionalized tips at both room temperature (RT) and low temperature (LT). We identify atomic contrast changes resulting either from a polarity change of the tip-terminating cluster or from a polarity-preserving tip change. Following the recently developed model for contrast formation on CaF_2_(111) surfaces [10], we adopt the contrast mode notations **C**_1_, **C**_3_, and **C**_4_* for a positively terminated tip and **C**_2_ and **C**_4_ for a negatively terminated tip. The distance-dependent contrast evolution [10] is summarized along the vertical columns in Figure 1. This figure additionally includes markers for tip changes as demonstrated in this work: Black solid arrows mark contrast changes exhibiting a change in tip polarity demonstrated in this work, while polarity-preserving tip changes are indicated by grey arrows. In addition, dashed arrows denote polarity changing tip changes that were observed during our experiments but are not discussed in the following as they represent the reverse direction of presented cases.

The contrast modes **C**_4_ or **C**_4_* are cyclic members of the same contrast mode, as introduced in [10]. Consequently, the assignment of NC-AFM image data to these contrasts modes requires the acquisition of systematic distance-dependent measurements [10]. Without such distance-dependent data, the contrast mode assignment is questionable.

## Experimental

RT experiments were performed on a bulk CaF_2_ crystal after preparing a clean CaF_2_(111) surface by cleaving the crystal in ultra-high vacuum (UHV) [23]. For the LT experiments, a CaF_2_/CaF/Si(111) thin film sample was used. The sample was prepared in situ by first degassing a p-type Si (B-doped) sample (Institute of Electronic Materials Technology, Warsaw, Poland) for several hours after introduction into the vacuum. Second, the Si(111)-(7 × 7) termination was formed by flash annealing cycles. Third, CaF_2_ material (99.9% purity) was deposited on the Si(111)-(7 × 7) surface from an EFM3T e-beam sublimator (Focus GmbH, Huenstetten, Germany) at substrates temperatures of 550 °C. Under these conditions, a CaF interface layer is formed, which removes the Si (7 × 7) reconstruction and allows for growing multilayers of well-ordered CaF_2_(111) [24–25], see [26] for further preparation details.

RT experiments were performed with a UHV 750 AFM system (RHK, Troy, MI USA) operated at a base pressure of 7.0 × 10^−11^ mbar. An Ar^+^ ion-sputtered silicon cantilever with an eigenfrequency of around 300 kHz and a quality factor of 22000 was used. The NC-AFM was operated in the frequency-modulation mode with an oscillation amplitude of 7.4 nm, and images shown herein were acquired in the quasi constant-height mode [18]. Frequency shift values printed in the respective images correspond to the setpoint of the feedback loop. LT experiments were performed at 77 K using a LT UHV STM/AFM (ScientaOmicron, Taunusstein, Germany) operated at a base pressure of 5 × 10^−10^ mbar. NC-AFM measurements were conducted with a quartz cantilever based on a tuning fork [27] and a chemically etched tungsten tip attached to the end of the active prong. The tip was further prepared in situ using common STM-based approaches on the bare Si surface after introducing the sensor into the vacuum system [28]. The NC-AFM microscope was operated in the frequency-modulation mode with an oscillation amplitude of 60 pm, and images were acquired in the true constant-height mode using an atom-tracking and feed-forward system for instantaneous drift compensation [29].

All frequency shift (Δ*f*) images are presented with regions of strong attractive tip–sample interaction depicted as ‘bright’ and regions of weak attractive or repulsive interaction reproduced as ‘dark’. In NC-AFM, the frequency shift Δ*f* is proportional to the weighted average of the tip–sample interaction force gradient [30]. Attractive forces mostly exhibiting a positive force gradient are considered as negative and yield a negative Δ*f* according to a generally accepted convention. When acquiring data in the constant height mode, we invert Δ*f* images so that a steeper force gradient appears as a brighter feature corresponding to an elevation in an image of the same feature taken in the constant frequency shift (topography) mode. Arrows in the upper right corner of Δ*f* images represent the fast (horizontal) and slow (vertical) scan directions.

The surface directions for the bulk crystal exposing the (111) surface can be determined by cleaving the crystal along another surface from the {111} family [31]. For CaF_2_ thin films grown on Si(111) surfaces, it has been established that the film grows in type-B epitaxy [24–25,32]. This implies that the 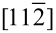 direction of the silicon crystal surface points in opposite direction of the 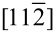 direction of the CaF_2_ thin film. The 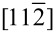 direction of the pristine Si(111) (7 × 7) surface was determined by identifying the faulted and unfaulted halves of the (7 × 7) reconstructed unit cell from STM imaging [33]. With the surface orientation established, the sublattices can be identified through a distance-dependent analysis of NC-AFM images [10], and corresponding model drawings of the CaF_2_(111) surface geometry are superimposed on the image data.

To improve the signal-to-noise ratio of the Δ*f* data in the RT experiments, unit cell averaging is performed as described in [10]. From the unit-cell averaged data, contrast profile lines Δ*f*_⟨uc⟩_ are extracted along the diagonal of the unit cell in the 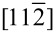 direction, with the resulting data included as traces next to the respective NC-AFM images.

## Results and Discussion

**Figure 2:**
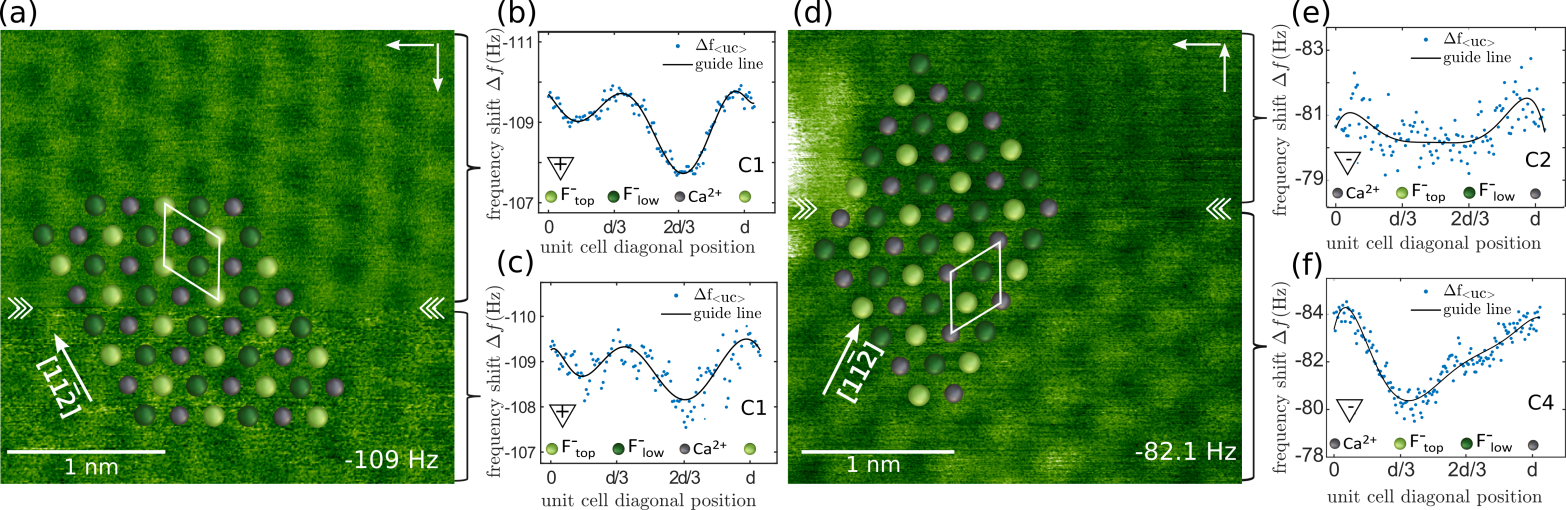
Examples of polarity-preserving tip changes on CaF_2_(111) at room temperature. (a, d) Δ*f* data acquired at (a) Δ*f*_set_ = −109 Hz and (d) −82.1 Hz with a top view CaF_2_(111) surface model overlaid. (b, c) and (e, f) Contrast profiles extracted along the 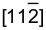 direction of the unit-cell averaged data from image regions indicated by the square brackets. 
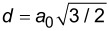
 represents the distance between equivalent atoms along ⟨11−2⟩ directions, where *a*_0_ is the bulk lattice constant of CaF_2_. Atomic assignment follows the model introduced in [10], with the solid line in panels (b, c, e, f) representing a polynomial fit of degree seven as a guide to the eye.

In Figure 2, we present RT data showing two examples of abrupt contrast changes where the polarity of the tip is maintained (polarity-preserving tip changes) but a different atomic contrast appears. The occurrences of tip changes are marked by the two chevron arrows framing the respective scan line. The nature of these changes as polarity preserving can be assessed from the contrast profiles shown in Figure 2b,c,e,f based on the conclusions in [10]. The image in Figure 2a maintains contrast mode **C**_1_, yet with an abrupt change in intensity (see Figure 2b,c), while the contrast change present in Figure 2d represents a transition from contrast mode **C**_4_ (see Figure 2f) to contrast mode **C**_2_ (see Figure 2e). The assignment of the contrast mode **C**_4_ follows a distance-dependent analysis of the data acquired prior to this image (data not shown). Positioning the CaF_2_(111) surface models in Figure 2a and Figure 2d relative to the NC-AFM data is based on the sublattice analysis of the contrast profiles shown in Figure 2b,c and Figure 2e,f, respectively. This positioning indicates that there is no lateral shift involved during the tip changes as the same lattice fits well before and after the tip changes. While this is true for the NC-AFM images shown here, images indicating a lateral shift upon a tip change are commonly observed. The assignment to a polarity-preserving tip change is based on the finding that for Figure 2a, the contrast mode **C**_1_ is related to a positively terminated tip, whereas for Figure 2d, the contrast modes **C**_2_ and **C**_4_ are both explained by a negatively terminated tip.

**Figure 3:**
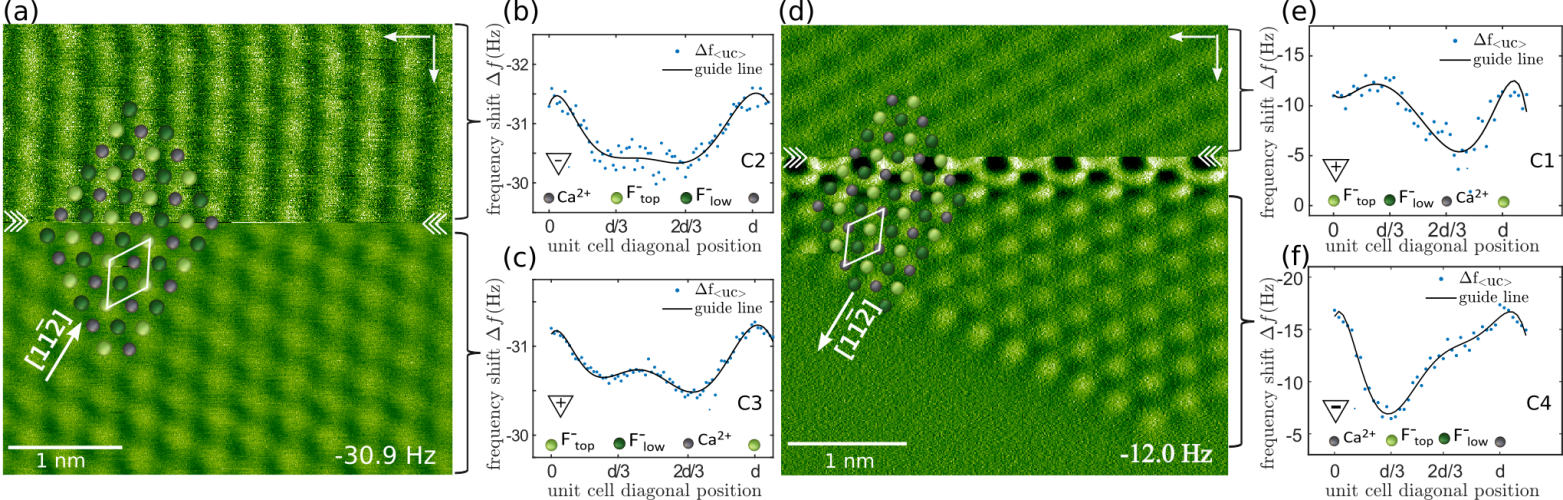
Examples of polarity-changing tip changes on CaF_2_(111). (a) Δ*f* data acquired at Δ*f*_set_ = −30.9 Hz (RT) and (d) Δ*f* data acquired on a thin film sample at Δ*f*_set_ = −12.0 Hz (77 K). Top-view CaF_2_(111) surface models are overlaid. (b, c) and (e, f) show line profiles extracted along the 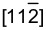 direction of the unit-cell averaged data in the regions indicated by the square brackets. 
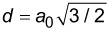
 represents the distance between equivalent atoms along ⟨11−2⟩ directions, where *a*_0_ is the bulk lattice constant of CaF_2_. Atomic assignment follows the model introduced in [10], with the solid line in panels (b, c, e, f) representing a polynomial fit of degree seven as a guide to the eye.

Next, we discuss tip changes that modify the tip polarity with exemplary data for negative-to-positive and positive-to-negative transitions reproduced, respectively, in Figure 3a and Figure 3d. In particular, the image data in Figure 3a recorded at RT and the corresponding contrast profiles (Figure 3b,c) exemplify a contrast change from **C**_2_ (associated with a negatively terminated tip) to **C**_3_ (associated with a positively terminated tip). In contrast, the image data in Figure 3d acquired at LT and the corresponding contrast profiles (Figure 3e,f) show an abrupt change from contrast mode **C**_1_ to **C**_4_, implying a change from a positive to a negative tip termination. To maintain stable imaging, the tip was retracted by about 100 pm immediately after the tip change, explaining the abrupt change in image contrast. Based on the sublattice identification in the contrast profiles in Figure 3b,c and Figure 3e,f, we superimpose the CaF_2_(111) surface model to the data in Figure 3a and Figure 3d and furthermore find that the tip change clearly goes along with a change in polarity of the contrast forming tip cluster in both cases.

A tentative explanation for the positive-to-negative tip change is a pickup of a fluorine ion from the surface by the tip, resulting in a negative tip termination. As a consequence, the tip interacts strongly attractively with the surface Ca^2+^ ions, explaining the contrast enhancement induced by the tip change.

During the analysis of 213 images acquired at RT, we observed 32 tip changes, with repeated evidence for polarity changes in both directions. Among these, 72% resulted in negatively terminated tips, while 28% ended in positively terminated tips. Across all 213 analyzed images, 67% exhibited contrasts associated with negatively terminated tips and 33% with positively terminated tips. This consistent trend suggests that negative tip termination is the more stable configuration when imaging fluorite surfaces.

**Figure 4:**
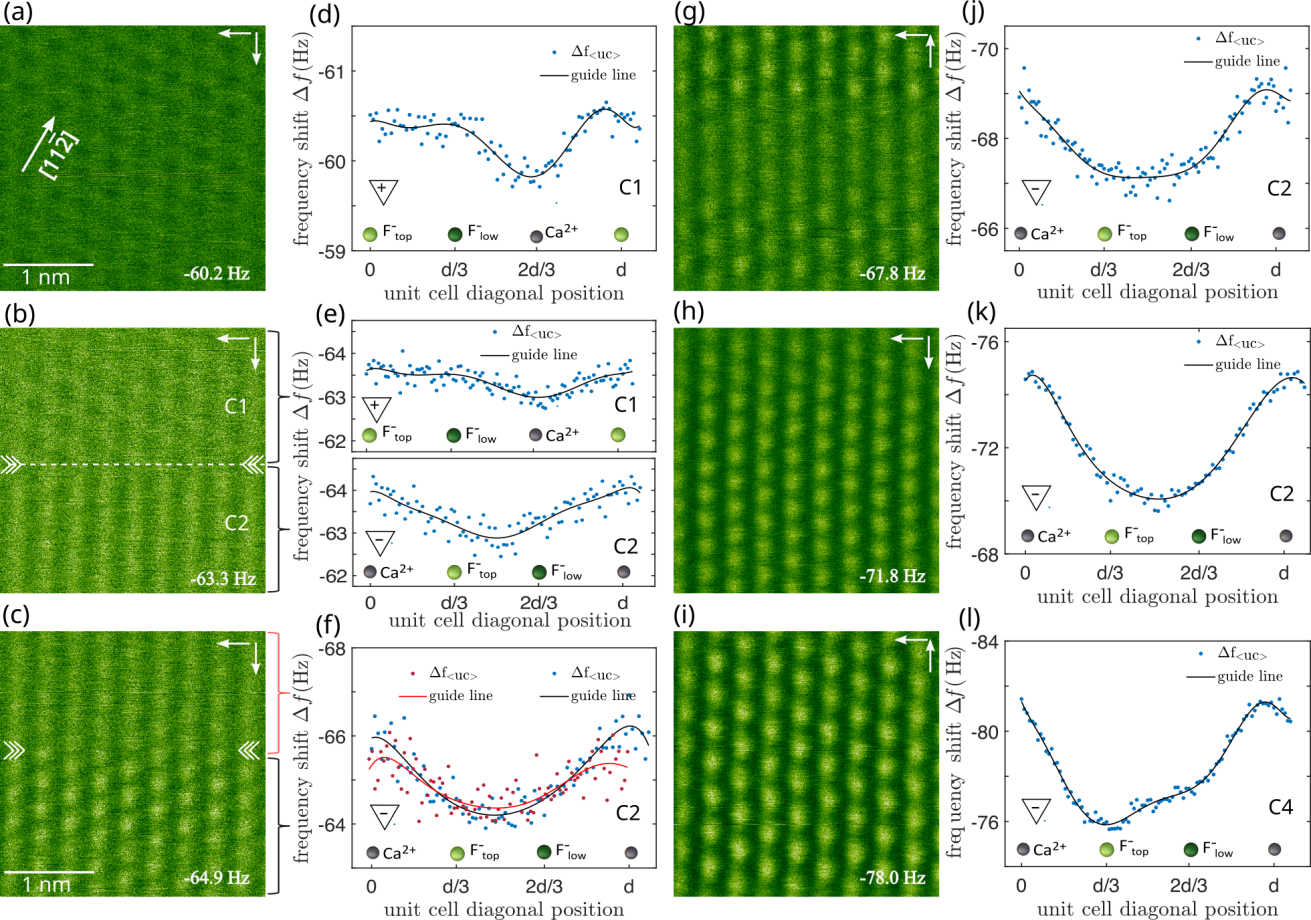
Tip changes leading to a successively stabilizing negative tip termination. (a–c) and (g–i) Δ*f* data acquired while step-wise decreasing the tip–sample distance (Δ*f*_set_ = −60.2 Hz to −78.0 Hz). (d–f) and (j–l) Line profiles extracted along the 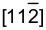 direction of the unit-cell averaged data. 
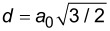
 represents the distance between equivalent atoms along ⟨11−2⟩ directions, where *a*_0_ is the bulk lattice constant of CaF_2_. Atomic assignment follows the model introduced in [10], with the solid line in panels (d–f) and (j–l) representing a polynomial fit of degree seven as a guide to the eye.

An intriguing example involving a sequence of tip changes to eventually arrive in contrast mode **C**_4_ is shown in Figure 4. Images in Figure 4a–c and Figure 4g–i represent image data acquired while step-wise decreasing the frequency shift setpoint. Such a reduction of the tip–surface distance eventually triggers tip changes. Contrast profiles for identifying the respective contrast modes are shown in Figure 4d–f and Figure 4j–l. It is found that the tip first yields contrast **C**_1_ (associated with a positively terminated tip) but experiences a polarity-changing tip change (Figure 4b) upon approach to the surface from contrast **C**_1_ to **C**_2_ (negatively terminated tip). Further approach reveals an unsteady **C**_2_ contrast (Figure 4c) as evidenced by the difference in contrast strength of the contrast profiles in Figure 4f, whereby the **C**_2_ contrast in the upper part (red contrast profile) is slightly weaker compared to that in the lower image half (blue contrast profile). A second polarity-preserving tip change is identified in the subsequent image in Figure 4g. Upon further decreasing the frequency shift setpoint, the **C**_2_ contrast stabilizes in Figure 4g to Figure 4h and eventually develops to contrast **C**_4_ (negatively terminated tip) in Figures 4i,l at further reduced tip–sample distance. While the evolution of contrast mode **C**_2_ to **C**_4_ is readily explained by the distance-dependence of imaging CaF_2_(111) with a negative tip [10], this series clearly shows the change from a previously positively terminated tip to a negatively terminated tip finally attaining a stable configuration.

Unlike in the RT data, where we observe both polarity-preserving and polarity-changing tip changes, at low temperature, so far no polarity-preserving tip changes were observed. This is a plausible result as tip stability is generally considered a merit of LT measurements. However, conclusions drawn from LT data are based on a much smaller number of measurements than those for RT data, and we anticipate that polarity-preserving tip changes at low temperature would be found as rare events in a sample of measurements with higher statistical significance.

## Conclusion

In conclusion, we present NC-AFM data demonstrating tip changes on a bulk CaF_2_(111) surface at room temperature and on a CaF_2_/CaF/Si(111) thin film surface at 77 K. We demonstrate the effect of tip changes on the contrast formation and find, as a key result, routes for a discrimination between polarity-preserving tip changes and tip changes associated with a change in tip polarity. Experimental evidence of both cases is found, with a tendency for negative tip termination to be the more stable configuration. We tentatively interpret this finding as a result of picking up a surface fluorine ion by the tip.

## Data Availability

Data generated and analyzed during this study is available from the corresponding author upon reasonable request.
